# 8C7: A Fully Human Anti‐PTGFRN Monoclonal Antibody‐Drug Conjugate Inhibiting Tumour Growth of Mesothelioma and Paediatric Medulloblastoma Cell Lines

**DOI:** 10.1111/jcmm.70665

**Published:** 2025-06-23

**Authors:** Jorge Marquez, Jianping Dong, Binbin Yue, Jun Hayashi, Chun Dong, Mitsuo Oshimura, Ginette Serrero

**Affiliations:** ^1^ Department of Pharmaceutical Sciences University of Maryland, Baltimore School of Pharmacy Baltimore Maryland USA; ^2^ Target Discovery Division A&G Pharmaceutical, Inc. Columbia Maryland USA; ^3^ Precision Antibody Columbia Maryland USA; ^4^ Trans Chromosomics, Inc. Yonago Tottori Japan

**Keywords:** antibody‐drug conjugate, medulloblastoma, mesothelioma, PTGFRN, xenograft

## Abstract

Antibody Drug Conjugates (ADCs) are attractive for developing cancer‐targeted therapies, particularly for cancers with unmet needs. Identification of a druggable internalising cell‐surface target enables the development of internalising monoclonal antibodies to deliver toxic payloads directly to the cancer cells. Using immunohistochemistry, we screened various non‐cancerous and cancerous tissue sections to assess PTGFRN expression levels. We produced hybridoma lines that produce fully human antibodies against the PTGFRN extracellular domain. After screening, we conjugated the cytotoxic payload Duocarmycin to an antibody candidate and tested its efficacy in in vitro assays, as well as in vivo xenografted athymic nude mice. We showed that PTGFRN expression was undetectable in non‐cancerous tissue samples and overexpressed in several patient‐derived cancer tissue samples. We produced a hybridoma line that produces a fully human IgG1 (8C7) against PTGFRN. 8C7 binds to cell‐surface PTGFRN, inducing endocytosis of PTGFRN. Direct conjugation of Duocarmycin to 8C7 resulted in an antibody‐drug conjugate that showed high potency in in vitro and in vivo models for three PTGFRN‐expressing cell lines examined, A431, DAOY, and MSTO, while it had no effect on PTGFRN‐negative MDA‐MB‐231. 8C7‐ADC administered via intraperitoneal injection to xenografted mice showed inhibition of tumour formation and growth with no effect on body weight and organ weights. These findings further validate PTGFRN as a target for antibody‐drug conjugate development for cancers with unmet needs.

**Abbreviations**
ADC: Antibody‐Drug Conjugate
BLI: Biolayer interferometry
DAR: drug antibody ratio
HIC: Hydrophobic Interaction Chromatography
IHC: Immunohistochemistry
MPM: Malignant Pleural Mesothelioma
OS: Overall Survival
PEG: Polyethylene Glycol
PFS: Progression‐Free Survival
PTGFRN: Prostaglandin F2 Receptor Negative
SD: Standard Deviation
SDS‐PAGE: sodium dodecyl sulfate polyacrylamide gel electrophoresis
TMA: Tissue microarray
TME: Tumour Microenvironment

## 1 Introduction

In oncology, Antibody‐Drug Conjugates (ADCs) have gained academic, regulatory, and industry attention due to their highly specified targeting capability, high tissue specificity, low off‐target effects, and ability to be conjugated to a variety of potent payloads [1]. ADCs are comprised of a monoclonal antibody recognising a unique cell‐surface antigen, a bridging chemical linker, and a cytotoxic drug payload. The large selection of linkers and payloads has the potential to be mixed and matched in order to optimise efficacy and minimise off‐target toxicity of the resulting ADC [2].

Most currently developed ADCs are either humanized, chimerized, or developed as fully human IgG [3, 4, 5]. Regardless of the method, the rationale is to reduce potential immunogenicity of the host, and the accompanying toxicity and enhancement of drug efficacy [6]. In most instances, the binding of the antibody to its cell surface target induces endocytosis to the cell interior, although in some ADC designs, this internalization step is not required for its mechanism of action [7].

The chemical linker that attaches the toxic payload to the antibody can be either cleavable (enzymatic, chemical, or physiochemical) or non‐cleavable and carry their own metabolic characteristics for half‐life, efficacy, and toxicity [8].

The selection of the cytotoxic payload is another consideration for the successful generation of a potent ADC. Payloads can be classified into one of two groups: 1) DNA‐damaging agents, or 2) microtubule inhibitors [9, 10]. It is believed that most ADCs are only capable of delivering 1%–2% of the administered dose, further highlighting the importance of the payload and its required potency [11].

However, a crucial part of developing ADCs is identifying a suitable target and developing a potent antibody to which these linkers and payloads are attached. HER2 receptor, Nectin‐4, Trop‐2, Tissue factor (TF‐011), and folate receptor (FRα) are all cancer cell surface proteins whose expression is significantly upregulated in their respective cancers, making them prime candidates leading to FDA‐approved ADC therapeutics [12, 13, 14, 15, 16].

Our laboratory had previously selected from an antibody library raised against cancer cells an antibody 33B7 able to bind and internalise into cancer cells. Two‐step immunoprecipitation followed by Mass spectrometry analysis identified the transmembrane protein Prostaglandin F2 Receptor Negative (PTGFRN) as the 33B7 target [17]. PTGFRN has been reported to be significantly overexpressed in several cancer types, making it an attractive target for ADC development as a cancer therapeutic. When comparing metastatic cancer cells to their non‐metastatic counterparts, PTGFRN mRNA and protein expression have been reported to be significantly upregulated in some lung cancers (NCI‐H460), melanomas (MDA‐MB‐435), and glioblastomas (U87MG and A172) [18, 19, 20, 21]. Additionally, PTGFRN expression was also found to have prognostic value for glioblastoma patients, and imparted a resistance to radiation sensitivity of these cancers [18]. Our laboratory has previously identified PTGFRN as being expressed and internalized in epidermoid carcinoma, paediatric medulloblastoma, and mesothelioma cancer cells [17]. Recently, our laboratory has reported how the expression of PTGFRN was positively correlated with a metastatic phenotype in cancer cells using both mass spectrometric proteome analysis, as well as cell‐based functional assays by examining the phenotypic changes in cells where PTGFRN expression had been silenced by SiRNA transfection, and conversely by examining the cellular changes associated with PTGFRN expression [22, 23]. This makes PTGFRN a valuable target for ADC development, as it is preferentially expressed in certain cancers compared to non‐cancerous tissue, it can act as a biomarker to predict response and stratify patients, and is found to be overexpressed when certain cancers become metastatic [24]. Additionally, to our knowledge, this ADC would be the first fully human therapeutic candidate, using PTGFRN as the target. There has been one other anti‐cancer therapy published involving PTGFRN‐expressing vesicles used to deliver a STING agonist to the tumour microenvironment (TME). In this case, the agonist would activate an immune response and recruit macrophages to the TME, as opposed to being an anti‐cancer agent itself [25].

In this present study, we used hybridoma technology with humanized transgenic mice to develop a fully human anti‐PTGFRN monoclonal antibody (8C7), when conjugated to the toxic payload Duocarmycin, shows efficacy in inhibiting tumour formation in xenograft mouse models of epidermoid carcinoma, paediatric medulloblastoma, and mesothelioma cancers. Further development of the 8C7‐ADC could provide a new line of therapeutic development for aggressive cancers with unmet needs.

## 2 Materials and Methods

### 2.1 Cell Lines

The human epidermoid carcinoma A431 (CRL‐1555), human paediatric medulloblastoma DAOY (HTB‐186), human biphasic mesothelioma MSTO‐211H (CRL‐2081), and human breast cancer MDA‐MB‐231 (CRM‐HTB‐26) cell lines were obtained from the American Type Culture Collection (ATCC, Manassas, VA). HEK‐293 cells overexpressing PTGFRN (HEK‐PTG) have been developed in our laboratory and described previously [17]. A431 cells and DAOY cells, where PTGFRN expression has been inhibited by shRNA transfection, and MSTO‐211H transfected with PTGFRN cDNA have been described previously [23]. These cells and their respective transfected clones were all cultured in a 1:1 mixture of Dulbecco's Modified Eagle Medium (DMEM)/Ham's F12 medium (DMEM/F12) supplemented with 50 μg/mL Gentamicin and 5% fetal bovine serum (FBS) in a humidified atmosphere 5% CO_2_ incubator at 37°C.

### 2.2 Development of Fully Human Anti‐PTGFRN Monoclonal Antibodies

The fully human anti‐human PTGFRN 8C7 antibody was generated in our laboratory by immunising humanised TC‐mAb mice [26] against his‐tagged recombinant protein containing the extracellular domain of human PTGFRN (His‐PTGFRN‐ECD). Five total injections were performed on the mice prior to fusion and hybridoma clone selection.

Humanized TC mice (TC‐mAb mice), which have been described previously [26], stably maintain a mouse‐derived engineered chromosome containing the entire human Ig heavy and kappa chain loci in a mouse Ig knockout background, leading to the development of fully human therapeutic monoclonal antibodies (Abs) when immunized with the antigen of interest. The advantage of directly developing fully human antibodies allows us to skip the humanization process needed for antibodies of mouse origin.

Single clone hybridomas were screened for binding to His‐tagged PTGFRN‐ECD by enzyme‐linked immunoassay (ELISA), as well as for binding to cells with high PTGFRN expression levels when compared to PTGFRN‐negative cells by Flow Cytometry. Finally, the hybridomas were screened for their ability to be internalised in PTGFRN‐positive cells compared to PTGFRN‐negative cells, followed by testing in killing assays of PTGFRN‐positive cells versus PTGFRN‐negative cells. The antibody affinity for binding to Human PTGFRN was determined by kinetics analysis using bilayer interferometry on Octet Red96 (Sartorius). The final selected 8C7 antibody was produced in serum‐free medium and purified by Protein A‐Sepharose chromatography. Gel electrophoresis and Coomassie blue staining in reducing and non‐reducing conditions were performed to confirm purity.

### 2.3 Flow Cytometry and Immunofluorescence to Determine Antibody Binding and Internalisation of PTGFRN

Flow cytometry analysis was used as a screening assay during the fully human monoclonal antibody development, comparing the binding ability of the hybridoma to HEK‐PTG versus HEK cells, and also to assay 8C7 antibody binding to A431, DAOY, and MSTO‐211H cells and their derivatives. For flow cytometry binding analysis, cells were detached with PBS‐5 mM EDTA. 5x10^5^ cells were incubated with increasing concentrations of human IgG isotype control or anti‐PTGFRN antibody 8C7 in DMEM + 1% BSA for 1 h at 4°C. Cells were washed with cold PBS three times and incubated with 20 μg/mL Goat‐anti‐Human IgG‐Alexa Fluor 647 (Jackson Immunoresearch #109–605‐088) in DMEM + 1% BSA for 1 h at 4°C. Subsequently, cells were washed with cold PBS three times, re‐suspended in PBS, and binding was measured using an Intellicyt Flow Cytometer (Intellicyt HTFC Screening System). For the MSTO‐211H cells flow binding study, a goat anti‐human‐red Phycoerythrin labelled (R‐PE) secondary antibody (Jackson Immunoresearch #109–116‐088) was used.

For Immunofluorescence analysis, chambered coverslips (Thermo Fisher #155380) were coated overnight at 4°C with 40 μg/mL type II rat collagen (Corning # 354236) in sterile deionised water. The next day, the collagen solution was aspirated from the coverslips and allowed to air dry for 2 h at room temperature. 1x10^5^ cells were seeded on coverslips overnight in a 37°C humidified 5% CO_2_ incubator. The next day, the cells were washed once with PBS and incubated with 1 μg/mL 8C7 or non‐immune human IgG antibody, both directly conjugated to Alexa Fluor647 diluted in 1% BSA in DMEM at 4°C for 1 h. The antibody solution also contained 1 mg/mL Hoechst 33,342 for nuclear staining. After binding incubation for an hour at 4°C, two coverslips were set in the 37°C incubator to initiate internalisation, with time points at 3 and 5 h. At each time point, the coverslips were washed three times with cold PBS and fixed in 4% paraformaldehyde for 10 min at room temperature. After PBS washing, the cells were mounted with ProLong Glass Antifade Mountant (Thermo Fisher #P36980) and viewed using a Nikon A1 point‐scanning laser confocal microscope (NIS‐Elements).

### 2.4 IHC to Screen Patient‐Derived Tissue Microarrays for PTGFRN Expression

The anti‐human PTGFRN mouse monoclonal antibody 1B4 was generated in our laboratory by immunizing mice with his‐tagged human PTGFRN ECD. Specificity to PTGFRN was shown by enzyme‐linked immunoassay, western blot analysis of recombinant PTGFRN protein, and by flow analysis of PTGFRN‐positive versus negative cells. ImmPRESS secondary antibody (MP‐7452) and ImmPACT DAB substrate kit (SK‐4105) were purchased from Vector Laboratories. Mayer's Haematoxylin was purchased from Millipore Sigma (MHS16). Background Terminator blocking solution was purchased from Biocare Medical.

Tissue microarrays containing multiple cases in duplicate or triplicate of cancerous and non‐cancerous sections of each tissue type analysed were purchased from TissueArray.com LLC.

Tissue array slides were deparaffinised by incubations in Xylene and rehydrated by successive washes with 100% and 95% ethanol, finished by incubation in distilled water. No Antigen Retrieval was required. Specimens were blocked with Background Terminator for 40 min at room temperature, then incubated with 1B4 antibody diluted in Antibody Enhancer Solution (AES) at 1 μg/mL for 30 min at room temperature (AES = 5% Goat Serum, 10 mM Glycine, 0.05% Tween20, 0.1% Triton X‐100, 0.1% H_2_O_2_).

The slides were washed three times with PBS containing 0.2% Tween 20 (PBS‐T) for 5 min each, then covered with a drop of ImmPRESS anti‐Mouse Polymer solution and incubated for 30 min at room temperature. The slides were washed with PBS‐T three times for 5 min each before adding a drop of ImmPACT Chromogen Substrate and incubating for 5 min. After washing them with water for 5 min, the slides were counterstained with Mayer's haematoxylin.

The slides were then dehydrated and mounted using ethanol and xylene, and images were captured using an Olympus BX40 microscope using TSView Version 7 software.

### 2.5 Antibody Conjugation to Duocarmycin

Tris(2‐carboxyethyl) phosphine (TCEP, 20490), L‐cysteine hydrochloride (L06328.22), and Dimethylacetamide (390800010) were all purchased from Thermo Fisher. The duocarmycin payload and valine‐citrulline linker moiety were purchased pre‐synthesised from MedChem Express in the form of MC‐Val‐Cit‐PAB‐Duocarmycin chloride (HY‐128904). The G‐25 gel filtration columns were purchased from Cytiva (17085101).

8C7 and Control antibodies were buffer exchanged into a conjugation buffer (pH 8) consisting of 25 mM Borate, 25 mM NaCl, and 1 mM Diethylenetriaminepentaacetic acid (DTPA), and concentrated to > 5 mg/mL. A 2.5 M equivalent of TCEP was added to the antibody solution and incubated for 40 min at 37°C. The antibody was immediately chilled on ice. 10% of Dimethylacetamide (DMA) was added to the antibody solution. MC‐Val‐Cit‐PAB‐Duocarmycin was added at a 1.2‐fold molar excess of the free thiol concentration and incubated at room temperature for 45 min. The reaction was quenched by adding a 20‐fold excess of Cysteine‐HCl (Millipore Sigma 30120) for 30‐min at room temperature. The ADC solution was buffer exchanged to completely remove unreacted Linker‐payload and free cysteine.

8C7‐Duocarmycin, as well as the Control Duocarmycin ADC, were purified on a pre‐packed HiScreen Butyl HP Hydrophobic chromatography (HIC) column (Cytiva Cat. 28978242) using a Bio‐Rad NGC Medium Pressure Chromatography System at room temperature. Before injection of the sample, the column was equilibrated with 3 column volumes of Mobile Phase A buffer (1.5 M Ammonium Sulphate, 25 mM Na_3_PO_4_, pH 8). The ADC was loaded onto the column and eluted using a linear gradient from 0% Mobile Phase B to 100% Mobile Phase B (25% Isopropanol, 25 mM Na_3_PO_4_, pH 8). The ADC fraction was collected, buffer exchanged to PBS (pH 7.4), and then sterilised by filtration through a 0.2 μm syringe filter. The antibody concentration was determined by Micro‐BCA. Ellman's Assay analysis was performed as an indirect determination of the drug antibody ratio (DAR) [27] according to the manufacturer's instructions (Thermo Fisher 22582) and showed the average drug to antibody ratio (DAR) of 4.

### 2.6 In Vitro Killing Assay to Test ADC Activity

In a 96‐well plate, 2000 cells/well for A431, DAOY, and MDA‐MB‐231 cells and 4000 cells/well for MSTO‐211H were plated in triplicate in DMEM/F12 medium supplemented with 5% FBS. 8C7‐ADC was added to a final concentration of 10 nM, 1 nM, 0.1 nM, and 0.01 nM. The control ADC was tested at the highest concentration of 10 nM.

After 72 h, cell proliferation was measured by CellTiter‐Glo Luminescent Cell Viability Assay reagent (Promega, Cat. G7572) according to the manufacturer's instructions. The luminescence levels were measured with a LMax II Luminometer (Molecular Devices, San Jose, CA). All experiments were carried out at least twice.

### 2.7 In Vivo Mouse Xenografts to Test ADC Effect on Tumour Growth

Animal studies were conducted in A&G's AAALAC‐accredited, OLAW‐certified animal facility. All mice housed in A&G Pharmaceutical's animal rooms were tested quarterly using a full PCR Rodent Infectious Agent (PRIA) panel, as well as skin and fur mite testing, done by Charles River. The protocols were reviewed and approved by A&G's IACUC animal committee. The effect of 8C7‐ADC and control‐ADC on tumour growth was examined with A431, DAOY, MSTO‐211H, and MDA‐MB‐231 cell lines. For each cell line, 30 mice were subcutaneously injected with 10^6^ cells/mouse in serum‐free DMEM/F_12_ medium. For A431 cells, 5x10^5^ cells were injected per mouse. The mice were monitored until the subcutaneous tumour reached approximately 50–100 mm^3^. Mice were randomised into three experimental groups and ear‐marked for individual identification. Animals with tumours that were too large or too small at the time of randomization were not included in the experiments. The number of mice per group was determined by power calculation based on the hypothesis of at least a 50% difference in tumour volume between the test and control groups [27].

For experiments with A431 and MSTO‐211H, the three experimental groups of 7 mice each were Control ADC (4.0 mg/kg), 8C7‐ADC (1.0 mg/kg), and 8C7‐ADC (4.0 mg/kg). For DAOY, ADC doses were 0.4 mg/kg and 1.6 mg/kg. For MDA‐MB‐231 cells, the Control ADC and 8C7‐ADC doses were 4.0 mg/kg. The ADCs were administered via intraperitoneal (IP) injection once weekly.

Mice were monitored daily for general health, signs of distress, and/or tumour necrosis. Mice were euthanised whenever tumour ulceration/necrosis was observed, or if mice were ill.

Tumour measurements were taken twice a week aseptically with a calliper, and volume expressed in mm^3^ was calculated using the formula (L x (W^2^)/2). Tumour volume increase was recorded as the tumour volume on the day of measurement divided by the initial tumour volume on Day 1. At the end of the experiments, the mice were weighed for final body weight and then euthanised following approved guidelines by isoflurane anaesthesia, exsanguination by cardiac puncture, followed by cervical dislocation. Tumours and major organs such as the liver, lungs, kidneys, and hearts were harvested from all mice and weighed. Sections of these organs were also fixed in formalin for 24 h, dehydrated in ethanol, and embedded in paraffin wax. These paraffin blocks were then sectioned for H&E staining and observed by a trained pathologist for any signs of cellular toxicity. All experiments were repeated twice.

### 2.8 Statistical Analysis

All flow cytometry and in vitro internalisation assays were performed in triplicate and repeated 3 times. Analysis of statistical significance was done using a Welsh's *t*‐test. Animal studies (*n* = 7) were analysed using Two‐Way Analysis of Variance (ANOVA) utilising post hoc Bonferroni analysis and/or Mixed Effects analysis.

## 3 Results

### 3.1 PTGFRN Is Highly Expressed in Several Tumour Tissue Types Including Mesothelioma and Medulloblastoma, but Not in Non‐Cancerous Tissue

**FIGURE 1:**
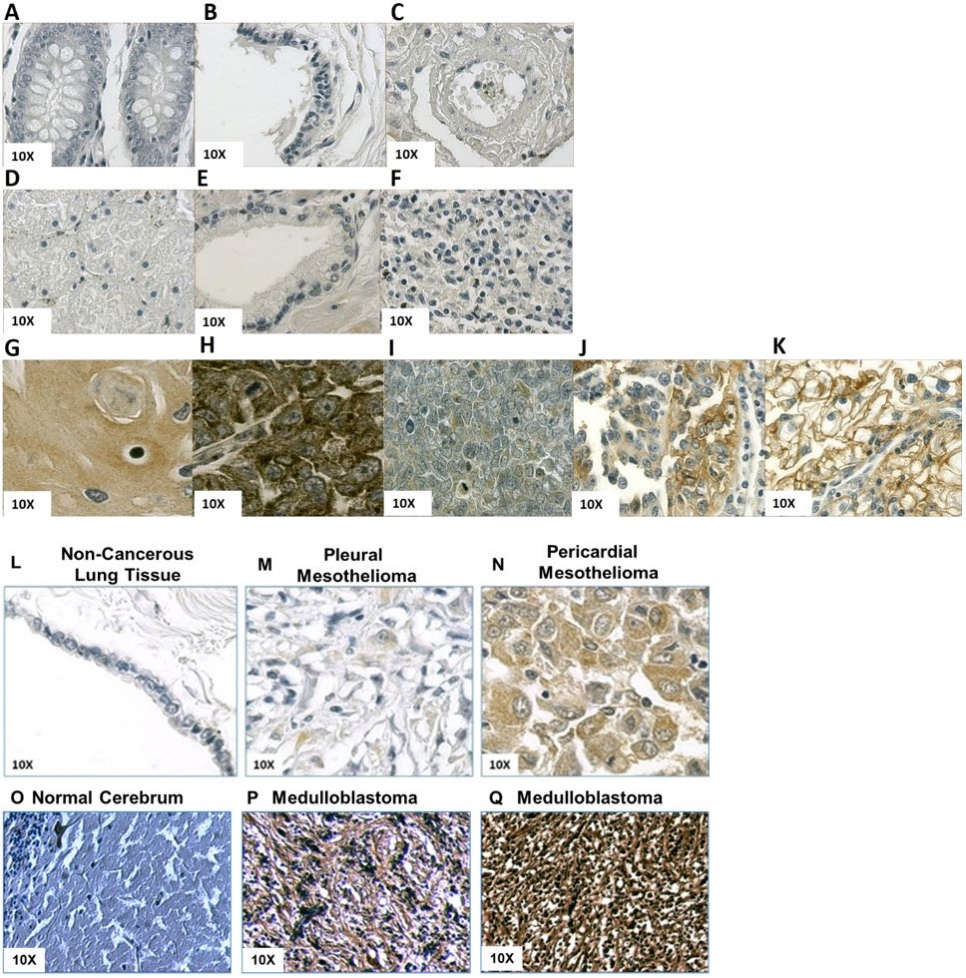
Immunohistological Analysis of PTGFRN Expression in Healthy and Cancerous Tissue Sections. Tissue microarrays were stained using 1 μg/mL of the anti‐PTGFRN IHC antibody 1B4 (brown) to assess the expression level of PTGFRN, as well as counter‐stained with haematoxylin for nuclear staining (blue). (A–K) Healthy non‐cancerous tissue, such as (A) Colon, (B) Breast, (C) Lung, (D) Kidney, (E) Prostate, and (F) Spleen, has no detectable levels of PTGFRN, whereas cancerous tissue samples from (G) Squamous Cell Carcinoma, (H) Melanoma, (I) Pancreatic Duct Adenocarcinoma, (J) Papillary Renal Cell Carcinoma, and (K) Clear Cell Carcinoma all show strong brown staining, indicating PTGFRN expression. (L–N) PTGFRN expression was examined in noncancerous lung tissue (L), pleural mesothelioma (M) and pericardial mesothelioma (N). (O–Q) PTGFRN expression was examined in tissue sections of normal cerebrum (O) and of cerebrum medulloblastoma (P) and medulloblastoma of the posterior cranium (Q).

Cell surface‐protein expression that differs between normal and cancerous tissue, or is cancer type‐specific, offers an attractive strategy for targeting cancers in a specific manner. Here, we examined PTGFRN expression in cancerous and non‐cancerous FFPE tissue sections by immunohistochemistry. The PTGFRN expression was undetectable in healthy tissues evaluated (Figure 1A–F). When screening various head & neck and kidney cancer tissue samples, Squamous Cell Carcinoma, Melanoma, Papillary Renal Cell Carcinoma, Clear Cell Carcinoma, and Pancreatic Duct Adenocarcinoma showed a strongly detectable level of PTGFRN expression (Figure 1G–K).

Among the cancer tissues examined, we investigated PTGFRN expression in mesothelioma and in medulloblastoma, which are two cancers with unmet needs for targeted therapies.

Since we had previously shown that PTGFRN was expressed in the human mesothelioma cell line MSTO‐211H and in the human medulloblastoma cell line DAOY [23], we examined PTGFRN expression by IHC in tissue sections from patient‐derived mesothelioma and medulloblastoma tissue arrays. In normal lung pleura tissue, PTGFRN expression was undetectable. Pleural mesothelioma tissue showed modestly elevated expression of PTGFRN, whereas pericardial mesothelioma tissue, which is commonly found when pleural mesothelioma metastasises, showed a significant increase in PTGFRN expression (Figure 1L–N).

In normal cerebrum tissue, PTGFRN expression was mostly negative (Figure 1O). Medulloblastoma tissue sections showed various degrees of PTGFRN expression from medium (Figure 1P) to very strong expression (Figure 1Q).

### 3.2 8C7 Is a Fully Human Antibody Binding Cell‐Surface PTGFRN, and Internalising the Transmembrane Protein

We developed a fully human anti‐PTGFRN antibody capable of internalizing the target protein. The details of the antibody development are described in the supplemental information section. By immunising humanized TC mice against the extracellular domain of PTGFRN, a library of single clone hybridomas was generated and screened to select fully human antibodies that bind to cell‐surface PTGFRN, are internalizing, and have a high affinity. Among the fully human anti‐PTGFRN antibodies developed, the anti‐PTGFRN antibody 8C7 was selected for its internalizing properties, and its K_D_ of 2.3 x10^−10^ M as determined by BLI with Octet Red96. The 8C7 antibody is an IgG1, considered to be a favourable isotype for ADC development [28]. Table 1 verified 8C7 binding to the cell surface of A431 and DAOY cells. It also showed that 8C7 binding was significantly decreased in A431 and DAOY cells after transfection with shRNA to knock down PTGFRN expression. In contrast, 8C7 binding levels increased in MSTO‐211H cells after transfection with human PTGFRN cDNA. Finally, no 8C7 binding was observed with the PTGFRN negative MDA‐MB‐231 cells when compared to the negative control antibody (Table 1). The shRNA‐ A431 and DAOY cell lines and cDNA‐transfected MSTO‐211H cell line used in these experiments had been previously characterised [23].

**TABLE 1:** Flow Cytometry assessing the specific binding of 8C7 to PTGFRN.

	A431		DAOY		MSTO‐211H		MDA‐MB‐231
	Control shRNA	PTGFRN shRNA	Control shRNA	PTGFRN shRNA	Empty vector	PTGFRN+	
hIgG MFI (1 μg/mL)	1034 ± 293	1632 ± 638	1757 ± 370	1169 ± 239	1362 ± 257	1411 ± 351	1308 ± 196
8C7 MFI (1 μg/mL)	577,616 ± 2322	31,548 ± 783	252,825 ± 3045	29,087 ± 2962	46,521 ± 3423	432,026 ± 4273	1517 ± 368
8C7 MFI (0.1 μg/mL)	105,012 ± 3549	10,682 ± 694	54,845 ± 4992	6037 ± 876	9742 ± 317	77,909 ± 2586	1287 ± 299

*Note:* 8C7 Flow Cytometry binding on A431, DAOY, and MSTO‐211H cells, as well as their PTGFRN +/− counterparts and on the PTGFRN‐negative breast cancer MDA‐MB‐231 cells. Results are presented as mean fluorescence intensity ± SD (*n* = 3).

**FIGURE 2:**
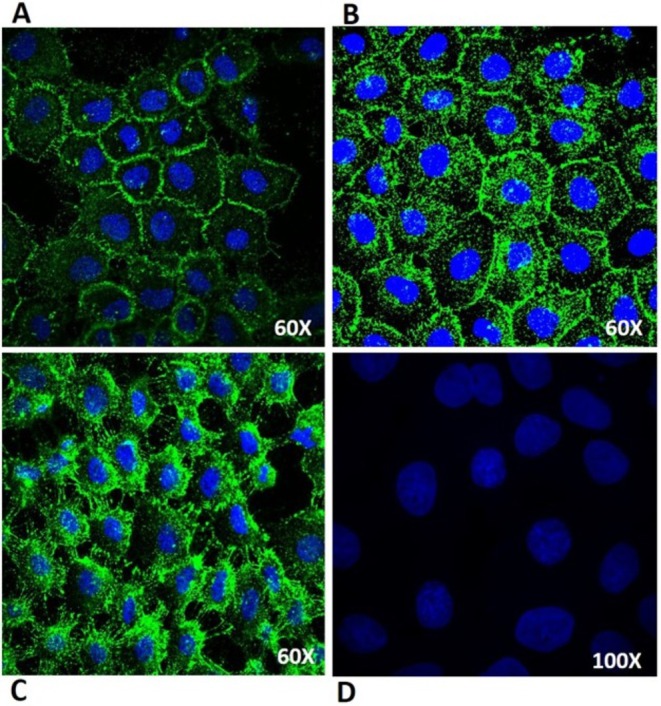
Internalisation of PTGFRN by 8C7. Binding of 8C7 antibody to cell‐surface PTGFRN in A431 cells induces endocytosis of the receptor. A431 cells were plated and incubated with 1 μg/mL 8C7‐Alexa Fluor 647 (Green) and Hoechst 33342 (blue) for 1 h at 4°C, followed by PBS washes, fixation in 4% paraformaldehyde, mounting, and imaging (time = 0) (A). The remaining cells were then incubated at 37°C for (B) 3 h, and (C) 5 h, and processed as described above. (D) Non‐immune human IgG‐Alexa 647 was used as a negative control. The cells in D were imaged at 100× to confirm the lack of fluorescent signal.

Immunofluorescence experiments were conducted to examine endocytosis of PTGFRN following 8C7 binding. As shown in Figure 2, the majority of 8C7 fluorescence was localised to the cell membrane and cell–cell junctions at time = 0. After 3 h of incubation at 37°C, the 8C7 immunofluorescence was observed intracellularly, with some of the signal still residing on the cell membranes. After 5 h incubation at 37°C, 8C7 fluorescence was seen primarily inside the cell, with little to no signal seen on the cell surface, confirming that PTGFRN is internalised following 8C7 antibody binding. Cells stained with a fluorescent hIgG negative control antibody showed no fluorescence signal other than the nuclear stain (Figure 4). Since 8C7 is a PTGFRN internalising antibody, its ability to deliver a cytotoxic payload to cells was examinednext.

### 3.3 Effect of 8C7‐Duocarmycin‐ADC in Vitro and In Vivo Models

**FIGURE 3:**
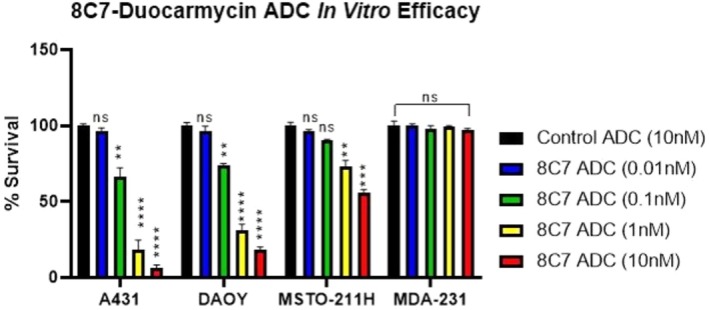
In Vitro Activity of 8C7‐Duocarmycin ADC. PTGFRN‐positive and negative cell lines were treated for 72 h with 8C7‐ADC at increasing concentrations (0.01 to 10 nM) or with 10 nM control ADC as a negative control. Experiments were done in triplicate and were repeated twice. Statistical significance was determined by Welsh's t‐test. Error bars are representative of standard deviation (**p < 0.005; ****p < 0.00005).

Duocarmycin is an anti‐cancer agent that exerts its effect by binding to the minor groove of the DNA double helix and alkylating the nucleotide adenine [29]. This binding and alkylation trigger tumour cell death. Conjugation of duocarmycin with a pre‐synthesised Duocarmycin‐Val‐Cit cleavable linker to 8C7 and control monoclonal antibodies was performed as described in the method section. The resulting antibody drug conjugate (8C7‐ADC) was first examined in vitro for its ability to inhibit the proliferation of PTGFRN‐expressing cell lines such as epidermoid carcinoma (A431), biphasic mesothelioma (MSTO‐211H), and paediatric medulloblastoma (DAOY). In addition, we also tested the ADC on the PTGFRN‐negative MDA‐MB‐231 cell line. As shown in Figure 3, 8C7‐ADC treatment showed a dose‐dependent inhibition of cell proliferation in all cell lines expressing PTGFRN. Specifically, A431 proliferation was reduced by 95% (IC_50_ = 0.25 nM), DAOY proliferation was reduced by 80% (IC_50_ = 0.47 nM), and MSTO‐211H proliferation was reduced by approximately 40% at the highest dose assayed of 10 nM. Control‐ADC, tested at the highest concentration of 10 nM, had no effect on the proliferation of all cell lines. The PTGFRN‐negative MDA‐MB‐231 cells showed no growth inhibition when treated with 8C7‐ADC, even at the highest concentration evaluated, indicating the specificity of the 8C7‐ADC for PTGFRN‐expressing cells.

We then examined the effect of 8C7‐ADC on tumour growth using in vivo xenograft models. The same cell lines used for the in vitro assays were subcutaneously injected into athymic nude mice. For each cell line, once the mice developed tumours, they were randomised into control and treatment groups. Two doses of 8C7‐ADC were evaluated to determine 8C7‐ADC treatment dose dependency. We observed a dose‐dependent and statistically significant reduction in tumour growth rate for the three PTGFRN‐expressing cells bearing mice treated with the 8C7‐ADC compared to the control ADC.

**FIGURE 4:**
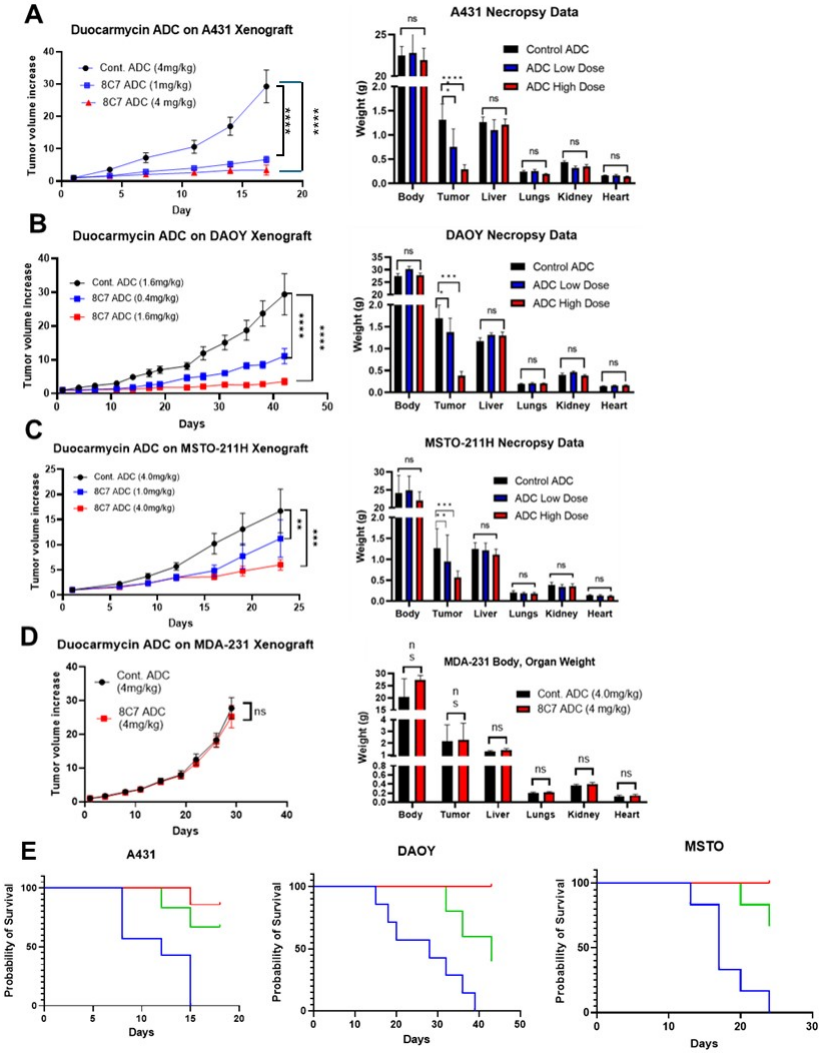
Effect of 8C7 ADC on tumour growth. Athymic nude mice were subcutaneously injected with A431 cells (A), DAOY cells (B), MSTO cells (C), and MDA‐MB‐231 (MDA‐231) (D). Both ADCs were administered by weekly intraperitoneal injections, with 3 ADC injections for A431 and MSTO‐211H and 5 ADC injections for DAOY and MDA‐231. (E) Survival curves for control ADC treatment (Blue line), 8C7 low dose (green line), and 8C7 high dose (red line) for A431, DAOY, and MSTO‐211H cells, respectively. Statistical significance was determined by Two‐Way Analysis of Variance (ANOVA) utilising post hoc Bonferroni analysis and/or Mixed Effect analysis. Error bars are representative of standard error (**p < 0.005; ***p < 0.0005; ****p < 0.00005).

We examined the effect of 1 mg/kg and 4 mg/kg of 8C7‐ADC on A431 tumour growth. Control ADC was used at 4 mg/kg. We had shown previously that when compared to PBS as a vehicle control, control‐ADC had no effect on tumour growth. Both 8C7‐ADC doses maximally inhibited A431 tumour growth by 80% and 90%, respectively (Figure 4A). We then examined the effect of 8C7‐ADC (0.4 mg/kg and 1.6 mg/kg) on the tumour growth of DAOY cells, which also displayed high expression of PTGFRN. Treatment with 1.6 mg/kg resulted in a tumour growth reduction of 85% in the DAOY tumours, while 0.4 mg/kg resulted in a tumour growth inhibition of 65% (Figure 4B).

MSTO‐211H cells, which express lower levels of PTGFRN, were treated with 8C7‐ADC at 1 and 4 mg/kg, resulting in a dose‐dependent tumour inhibition of 32% and 63%, respectively (Figure 4C). The PTGFRN‐negative cell line, MDA‐MB‐231, showed no response to the 8C7‐ADC. No difference in tumour growth was observed between the IgG negative control ADC and the 8C7‐ADC groups (Figure 4D). Figure 4E shows the tumour growth data represented as Kaplan– Meier graphs for each cell line examined. There was no decrease in body weight for the 8C7‐ADC‐treated animals compared to controls. There was no change in organ weights of treated animals compared to controls for all xenograft experiments, indicating the on‐target effect of 8C7‐ADC on tumour growth.

Pathological analysis of the organ tissues harvested from control and treated mice showed no signs of toxicity. No necrosis was observed in any of the tissues collected from mice.

Since PTGFRN is expressed in certain cancers with no expression in normal tissues, these results indicate that PTGFRN could be a valuable new ADC target as PTGFRN negative cells showed no response to the 8C7‐ADC, while tumours that express low level of PTGFRN, such as the MSTO‐211H tumours, still demonstrated a statistically significant response to the 8C7‐ADC. Tumours that displayed high PTGFRN expression, such as A431 and DAOY tumours, exhibited a high reduction in tumour growth with no obvious signs of off‐target toxicity in response to the 8C7‐ADC, demonstrating its efficacy.

## 4 Discussion

Paediatric medulloblastoma and mesothelioma are rare and aggressive cancers with poor prognosis and unmet needs, which would benefit from the identification of druggable biological targets and the development of targeted antibody therapeutics [30, 31]. Mesothelioma typically arises in the lung pleura and occasionally in the peritoneum, as well as the pericardium and testes in some rare cases. Diagnosis is difficult, lacking routine blood tests. Mesothelioma treatment is complex, relying on surgery and a combination of chemotherapies, having limited effects and significant toxicities [32, 33, 34, 35, 36, 37]. Targeted therapy is limited to anti‐folate, anti‐VEGF antibody and the recent approval by the FDA of nivolumab and ipilimumab with improved OS and PFS [38].

Medulloblastoma is a central nervous system cancer that represents 25% of all paediatric brain neoplasms with a 5‐year survival rate of 72.1% [39, 40].

Current medulloblastoma treatment typically relies on surgery followed by postoperative radiation, as well as general chemotherapy, with no specific chemotherapeutic agent that has demonstrated superiority in treating medulloblastoma [41, 42].

These two rare and aggressive cancers display unmet needs in terms of targeted therapies. The identification of PTGFRN as an ADC target is the first step in developing specific therapy for solid tumour cancers that express PTGFRN, such as medulloblastoma and mesothelioma.

The immunohistochemistry we performed indicated that PTGFRN expression was undetectable in various non‐cancerous tissues, whereas PTGFRN expression was significantly elevated in several cancers, including mesothelioma and medulloblastoma. Interestingly, PTGFRN IHC in patients' samples showed negative staining in a healthy lung sample, mild PTGFRN expression in a pleural tumour, but significantly increased PTGFRN staining after invasion of the pericardium. These results are interesting in the light of studies including ours [23] demonstrating that PTGFRN expression is associated with increased metastatic properties. We had shown previously that inhibition of PTGFRN expression in A431 cells and in DAOY cells inhibited cell proliferation in low serum, migration, clonogenicity, and Spheroid formation in 3D culture, all hallmarks of cancer aggressiveness [23]. Conversely, overexpression of PTGFRN in mesothelioma MSTO‐211H resulted in an increase in proliferation, migration, clonogenicity, and Spheroid formation in 3D culture [23]. An ADC that targets increased expression in advanced lesions of a biomarker like PTGFRN in mesothelioma would be an invaluable tool to develop new treatments for patients suffering from these advanced‐stage cancers, along with the availability of a biomarker to identify patients [43].

Conjugation of the fully human anti‐PTGFRN antibody 8C7 to the payload Duocarmycin using a cleavable valine‐citrulline linker resulted in an ADC that demonstrated high efficacy in inhibiting cancer cell proliferation in in vitro models. Furthermore, this same ADC also displays efficacy in reducing tumour growth in vivo. Our 8C7‐ADC was found to be selective as well, with no effect seen in a PTGFRN‐negative cell line, such as MDA‐MB‐231. Comparison of body and organ weights between the control and 8C7‐ADC experimental groups at the end of all xenograft experiments showed no significant differences in any weights between the 2–3 groups. This would suggest that there is little off‐target toxicity or inflammation associated with the administration of 8C7‐ADC. As mentioned previously, these organ tissue sections were also histopathologically analysed, and no signs of significant inflammation or necrosis were reported.

These results enhance the ones obtained in our previous study with 33B7 mouse monoclonal antibody, which was selected from a cancer cell hybridoma library and used to identify PTGFRN as a cancer target [17]. Treatment of mice bearing A431 tumours with a 33B7‐Saporin conjugate showed no obvious signs of off‐target toxicity to any mouse organs, supporting PTGFRN's potential as a viable ADC cancer target [17].

While our ADC in its current form showed impressive therapeutic efficacy, there is still room for improvement. MMAE, exatecan, PNU159682, and DM‐1 were all screened alongside duocarmycin when initially considering possible payloads. Ultimately, the superior in vitro potency of duocarmycin as a payload, which has been reported for several other ADCs [44, 45, 46, 47, 48, 49, 50, 51, 52] resulted in it being selected as the final payload for these experiments.

Additionally, it may be beneficial to examine the effect of 8C7‐ADC with Patient‐Derived Xenograft (PDX), as they offer advantages compared to cultured cancer cell lines [53, 54]. These experiments are underway. The reported results present 8C7‐ADC as a novel therapeutic opportunity for aggressive cancers, such as paediatric medulloblastoma and mesothelioma, an area of unmet need.

## Author Contributions

J.M. and G.S.: literature research, study concepts and design, experimental studies, guarantor of integrity of the entire study, statistical analysis, manuscript preparation. J.M., J.D., B.Y., and C.D.: experimental studies. J.H. and O.M.: transgenic mice and hybridoma development. All authors have read and agreed to the published version of the manuscript.

## Ethics Statement

This study is reported in accordance with ARRIVE guidelines. Xenograft studies were conducted in strict accordance with the recommendations of the Guide for the Care and Use of Laboratory Animals from the National Institutes of Health. The protocol used for these studies was approved by the Institutional Animal Care and Use Committee (IACUC) of A&G Pharmaceutical (Protocol number: GS‐01). All animal procedures were performed under isoflurane anaesthesia, and all efforts were made to minimise animal suffering. 4–6‐week‐old female athymic nude mice (Charles River) were housed aseptically with up to 5 mice/cage in the Institution's AAALAC‐accredited and OLAW‐certified animal facility (OLAW D16‐00700).

## Conflicts of Interest

All authors except Jorge Marquez and Mitsuo Oshimura are employees of A&G Pharmaceutical Inc. Jorge Marquez and Mitsuo Oshimura have no conflicts.

## Data Availability

All data generated or analysed during this study are included in this published article. Data and materials were generated by the authors and are available on request. The datasets used and/or analysed during the current study are available from the corresponding author on reasonable request.
